# Endothelial Dysfunction and Diabetes: Effects on Angiogenesis, Vascular Remodeling, and Wound Healing

**DOI:** 10.1155/2012/918267

**Published:** 2012-02-12

**Authors:** Gopi Krishna Kolluru, Shyamal C. Bir, Christopher G. Kevil

**Affiliations:** Department of Pathology, LSU Health Sciences Center-Shreveport, 1501 Kings Highway, Shreveport, LA 71130, USA

## Abstract

Diabetes mellitus (DM) is a chronic metabolic disorder characterized by inappropriate hyperglycemia due to lack of or resistance to insulin. Patients with DM are frequently afflicted with ischemic vascular disease or wound healing defect. It is well known that type 2 DM causes amplification of the atherosclerotic process, endothelial cell dysfunction, glycosylation of extracellular matrix proteins, and vascular denervation. These complications ultimately lead to impairment of neovascularization and diabetic wound healing. Therapeutic angiogenesis remains an attractive treatment modality for chronic ischemic disorders including PAD and/or diabetic wound healing. Many experimental studies have identified better approaches for diabetic cardiovascular complications, however, successful clinical translation has been limited possibly due to the narrow therapeutic targets of these agents or the lack of rigorous evaluation of pathology and therapeutic mechanisms in experimental models of disease. This paper discusses the current body of evidence identifying endothelial dysfunction and impaired angiogenesis during diabetes.

## 1. Introduction

**Figure 1:**
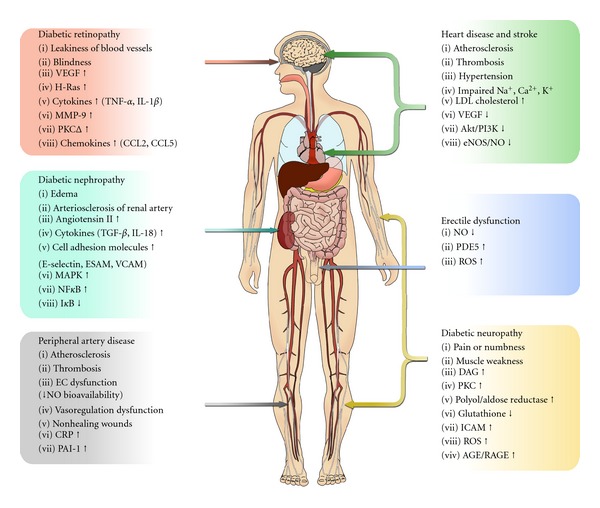
Diabetic vascular disease effects and symptoms. Various pathophysiological conditions affected in the body due to diabetic vascular disease are illustrated. Prominent symptoms of diabetes mediated abnormalities are indicated for each condition.

Endothelial cell dysfunction (ECD) is a broad term which implies dysregulation of endothelial cell functions, including impairment of the barrier functions of endothelial cells, vasodilation, disturbances in proliferative capacities, migratory as well as tube formation properties, angiogenic properties, attenuation of synthetic function, and deterrence of white blood cells from adhesion and diapedesis [1]. Several factors contribute to ECD including smoking, high blood pressure, diabetes, high cholesterol levels, obesity, hyperglycemia, advance glycation end products (AGEs), and genetic factors [1, 2]. Diabetes is a chronic metabolic disorder characterized by inappropriate hyperglycemia due to lack of or resistance to insulin, which contributes to ECD. About 170 million people worldwide are affected by diabetes including 20.8 million diabetic patients in the USA, numbers projected to double by 2030 [3]. Diabetes can be stratified into two groups with type 1 diabetes being insulin dependent and type II insulin independent. Both type 1 and type 2 cause hyperglycemia, which in turn causes endothelial dysfunction by its different glycooxidative products. Type 2 diabetes causes insulin resistance which is also responsible for endothelial dysfunction [4]. Obesity, which is individually a risk factor for EC dysfunction is also closely related to type 2 diabetes [5]. These two amplify the ECD more frequently. Angiogenesis or neovascularization is a global term which typically involves arteriogenesis and vasculogenesis [6]. These complex processes require multiple factors to stimulate vascular sprouting, remodeling, and recruitment of endothelial cells as well as establish stable vasculature [6, 7]. Angiogenic responses are known to be defective in some tissues (e.g., peripheral limbs) while enhanced in other tissues (e.g., retina) during diabetes [8]. Here, we discuss the contribution of endothelial dysfunction and subsequent aberrant angiogenic responses in diabetes.  Figure 1 illustrates several pathophysiological conditions under diabetes and the major subsequent symptoms associated (Figure 1).

## 2. Endothelial Dysfunction

Endothelial dysfunction is a systemic pathological condition which can be broadly defined as an imbalance between vasodilating and vasoconstricting substances produced by the endothelium or overall functions of the endothelium [2]. Normal functions of endothelial cells include production of nitric oxide (NO), regulation of platelet adhesion, coagulation, immune function, control of volume, and electrolyte content of the intravascular and extravascular spaces. Endothelial dysfunction is primarily due to reduction in NO bioavailabilty, and a marker for vascular health. Endothelial dysfunction can result from and/or contribute to several disease processes, as occurs in diabetes mellitus, hypercholesterolemia and hypertension, and also due to environmental factors, such as smoking tobacco products and exposure to air pollution [9].

Specifically, endothelial dysfunction is associated with reduced nitric oxide production, anticoagulant properties, increased platelet aggregation, increased expression of adhesion molecules, increased expression of chemokines and cytokines, and increased reactive oxygen species production from the endothelium [10]. These all play important roles in the development of diabetic vascular complications including atherosclerosis and other vascular pathologies. Importantly, endothelial dysfunction has been shown to be of prognostic significance in predicting vascular events [11, 12], so endothelial function testing may potentiate the detection of cardiovascular diseases such as myocardial infarction, peripheral vascular disease, ischemic stroke, and others [13, 14]. 

**Figure 2:**
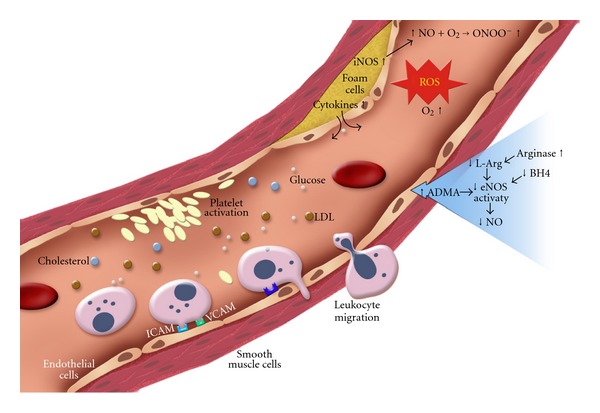
Hyperglycemic effects on the blood vessel. Atherosclerotic plaque formation initiated through uptake of LDL from blood by endothelial cells. Foam cells produce proinflammatory cytokines that are released into the lumen of blood vessel (far right). Increased ROS production through iNOS leads to increased ROS generation. Steps involved in leukocyte adhesion and migration (bottom left). Increased glucose leads to decreased L-arginine and BH4, which leads to decreased NO production in endothelial cells. All of these factors are proinflammatory and atherogenic.

An important feature of endothelial dysfunction is the inability of arteries and arterioles to optimally dilate in response to an appropriate stimulus by vasodilators acting on the endothelium. This endothelial dysfunction is notoriously associated with decreased NO bioavailability, which is due to impaired NO production by the endothelium and/or increased inactivation of NO by reactive oxygen species [15, 16].  Figure 2 illustrates the various steps involved in blood vessel leading to vascular endothelial dysfunction and inflammation under diabetes (Figure 2). Reduced NO bioavailability decreases the ability of endothelial cells to execute their functions in regulating vascular tone and growth, thrombosis, immune cell responses, and vascular barrier functions.

## 3. Diabetes

Diabetes mellitus is a group of metabolic diseases in which a person has high blood glucose either because the body does not produce enough insulin, or because cells do not respond to the insulin that is produced by the pancreas. This resulting high blood sugar produces the classical symptoms of polyuria: frequent urination polydipsia (increased thirst) and polyphagia (increased hunger) [17].

Type 1 diabetes results from the body's failure to produce insulin due to autoimmune or idiopathic destruction of cells, and may require the injection of insulin to control symptoms. In type 1 diabetes, the pancreas cannot synthesize enough insulin to maintain euglycemia. Type 1 diabetes is more common among children and young adults and insulin injections are used for treatment, thus type 1 diabetes is also referred to as insulin dependent diabetes mellitus (IDDM) or Juvenile Diabetes [17, 18].

In case of type II diabetes, there is normal production of insulin hormone but the body cells are resistant to insulin, a condition in which cells fail to use insulin properly, or sometimes combined with an absolute insulin deficiency. Cells and tissues are not responsive to insulin, so glucose remains elevated in the bloodstream. Type 2 diabetes is commonly manifested by middle-to-late-aged adults (40 years); however, its prevalence is increasing in younger populations. As insulin was initially not considered necessary for treatment of type 2 diabetes, it is known as non-insulin dependent diabetes mellitus (NIDDM) or Adult Onset Diabetes [17, 19].

A diabetic patient cannot metabolize carbohydrates, proteins or fats due to improper production of insulin, a blood glucose regulator, or resistance to insulin. Insulin helps cells use glucose as a main energy source. However, diabetic patients' cells do not make use of glucose from the blood due to abnormal insulin metabolism, resulting in elevated blood glucose levels or hyperglycemia. Over time, high glucose levels in the bloodstream can lead to severe complications such as vision loss, cardiovascular diseases, kidney disorder, and nerve damage [17–19].

## 4. Angiogenesis

Angiogenesis is a global term which indicates the physiological process involving the growth of new blood vessels or neovascularization. This is a vital process for embryological growth, tissue development, and wound healing in damaged tissues. Angiogenesis is also an important step in the transition of tumors from a confined locale to malignancy [20]. Neovascularization or angiogenesis has also been interchangeably associated with* vasculogenesis* which primarily refers to developmental formation of vascular structures from circulating or tissue-resident endothelial progenitor cells that proliferate into de novo endothelial cells. Angiogenesis predominantly relates to formation of endothelium-lined microvasculature with supportive cells (e.g., pericytes). Postembryonic vascular development plays an important role throughout life to address tissue metabolic and functional needs as well as reproductive physiological responses. Arteriogenesis refers to maturation and enlargement of smaller preexisting arterial vessels through vascular remodeling or collateral growth.

These processes require several biochemical and physiological factors to stimulate vessel sprouting and remodeling of the primitive vascular network, which in turn establish stable and functional blood vessel networks. There are several angiogenic factors which are involved in stimulation, promotion, and stabilization of new blood vessels such as VEGFs, FGFs, Angiopoietins, PDGF, MCP-1, TGF, various integrins, VE-cadherin, ephrins nitric oxide, and others [20–23]. Likewise mechanical stimulation such as physiological shear stress is also important particularly in arteriogenesis [24]. Angiogenesis and arteriogenesis/vascular remodeling represents excellent therapeutic options for the treatment of numerous cardiovascular diseases. Below is a table showing the causes of excessive and deficient angiogenesis under diabetes (Table 1).

### 4.1. Increased Production of Reactive Oxygen Species

Both endothelial cells and vascular smooth muscle cells are capable of producing reactive oxygen species from a variety of enzymatic sources. In disease states such as diabetes, vascular production of reactive oxygen metabolites can increase substantially [15]. Increased production of the superoxide anion (O_2_^−^) can lead to decreased tissue bioavailability of nitric oxide (NO) via a facile radical/radical reaction that occurs more rapidly than the reaction of O_2_^−^ with superoxide dismutase (SOD) [25]. This phenomenon alters endothelial regulation of vasomotion in a variety of disease conditions. Importantly, this endothelial dysfunction is due to vascular production of superoxide. There are several enzymes that involve generating ROS such as NADPH oxidase, aldehyde oxidase, xanthine oxidase, and glucose oxidase. Besides these, mitochondrial uncoupling also produces ROS by various mechanisms, which are all discussed below. 

#### 4.1.1. NADPH Oxidase

Recent evidence suggests that the major source of vascular superoxide ion and hydrogen peroxide (H_2_O_2_) occurs through membrane-bound, nicotinamide-adenine-dinucleotide- (NADH-) dependent oxidase (NOX) [26]. NOX is a transmembrane enzyme and generate superoxide by electron transfer from NADPH to molecular oxygen. The product of this reaction is the O_2_^−^, which undergoes secondary reactions. O_2_^−^ inactivates NO to yield peroxynitrite and can also spontaneously or under catalysis by SODs form H_2_O_2_ [27, 28]. NADPH-oxidase-derived ROS has been implicated in the regulation of vasodilation directly or indirectly by decreasing NO bioavailability [29, 30]. This observation provides a direct link between NADPH oxidase and endothelial function in humans. In pathological conditions such as diabetes, atherosclerosis, hypertension, cardiac failure, and ischemia reperfusion injury, ROS generation mediates endothelial cell dysfunction, cell proliferation, migration, inflammation, extracellular matrix deposition, fibrosis, angiogenesis, and cardiovascular remodeling [31, 32].

#### 4.1.2. Aldehyde Oxidases

Aldehyde oxidase (AO) produces O_2_^−^ and H_2_O_2_ from aldehyde [33, 34]. These aldehydes are the substrate for both AO and xanthine oxidase (XO). In diabetes, lipid peroxidation and glycation of protein are much more common than in normal healthy subjects [35]. Therefore, the contribution of oxidative stress from aldehyde AO would be higher. AO-derived ROS may also play role in cardiovascular complications in diabetes [36]. However, the pathophysiological importance of this pathway for diabetic vascular disease requires further study.

#### 4.1.3. Xanthine Oxidases (XOs)

Xanthine oxidase (XO) activity accounts for significant increased of ROS production in different tissues, as treatment with the XO inhibitor (allupurinol) reduces the ROS production [37]. Increase of both XO expression and activity results in imbalance of ATP/ADP ratio and increased ROS production in skeletal muscle of STZ-induced diabetic mice [38, 39]. These studies demonstrate that superoxide generated through increased XO seen in experimental diabetic mice is intimately involved in the pathogenesis of diabetic vascular complications.

#### 4.1.4. Glucose Oxidase

Glucose oxidase (GO) oxidizes glucose, which leads to production of free radicals resulting in oxidative stress during diabetes. There is evidence that glycation of protein depends on the oxidation of glucose since the product of glucose oxidation is attached to the protein to generate AGEs [40]. AGEs mediate increased NADPH subunit gp91 expression, NADH/NADPH oxidase activity, and decreased manganese super oxide dismutase (MnSOD) levels [41]. This finding indicates that AGEs play key pathophysiological roles in increasing oxidative stress in diabetes along with subsequent endothelial dysfunction through decreasing endothelial nitric oxide synthase (eNOS) phosphorylation.

#### 4.1.5. Mitochondrial Dysfunction

One of the major intracellular sources of ROS production is mitochondria. ROS formation is a byproduct of oxidative phosphorylation across the mitochondrial respiratory chain. Although mitochondrial complexes 1 and 3 are mainly responsible for generation of ROS, dysfunction of complexes 2 and 4 may result in electron leak and increased ROS production [32, 42]. Hyperglycemia in humans or animal models of diabetes is associated with impaired mitochondrial activity predominantly in vascular tissues resulting in mutations within mitochondrial DNA, ROS production, apoptosis, and endothelial dysfunction [43–45]. However, the vascular damage by mitochondrial ROS can be prevented by upregulation of MnSOD and UCP-1, which reduces PKC activation, formation of AGEs, and NF-*κ*B activation in endothelial cells [46].

#### 4.1.6. Comparison of ROS-Induced Pathogenesis and Subsequent Complications in Both Types of Diabetes

The combined effects of genetic susceptibility, environmental factors, and dietary deficiencies are known responsible for type 1 diabetes. Autoreactive T cells recognize and liberate ROS and proinflammatory cytokines [47]. Research-based evidence supported that there is an increase in ROS generation from activated phagocytes following viral attack [48]. NOX-derived as well as mitochondrial ROS have implications in *β* cell destruction and onset of diabetes. Hyperglycemia can increase assembly of NOX enzymes through its p47 phox subunit and therefore enhance superoxide production and facilitates *β* cell destruction [49]. The superoxide leaked from mitochondria can form H_2_O_2_ and work to uncouple glucose metabolism from insulin secretion. Ultimately, high level of oxidative stress can cause *β* cell death [46, 50]. 

Previously, Takasu et al. have demonstrated in rat model that alloxan induces type 1 diabetes which causes redox-mediated *β* cell DNA fragmentation culminating in cell death [51]. In contrast, recent studies show that alloxan resistant strain of mice shows increased ROS dissipation and resistance to *β* cell death [51]. Streptozotocin also produces XO-mediated superoxide that binds with NO to generate peroxynitrite [52]. Antioxidant defense of *β* cell mitochondria is exceptionally low due to its reduced glutathione peroxidase, SOD, and catalase activity [53]. This can make *β* cells vulnerable to excess oxidative stress and subsequent cytokine-mediated autoimmune attack [54]. Therefore, ROS whatever the source is directly or indirectly responsible for development of type 1 diabetes.

The hallmark of type 2 diabetes is insulin resistance as well as *β* cell dysfunction. ROS is involved in progression of insulin resistance, which leads to *β* cell dysfunction developing into type 2 diabetes [55, 56]. There are couples of mechanisms to ROS-induced onset of type 2 diabetes [57–64]. Tirosh et al. suggested that ROS disrupts insulin-induced cellular redistribution of insulin receptor substrate-1 (IRS-1) and phosphatidylinositol 3 kinase (PI3K) and thus impairing GLUT4 translocation in 3T3-L1 adipocytes [62]. Another mechanism is that hyperglycemia-induced excess ROS presumably lead to activation of JNK pathway. This activation of JNK pathway activates inflammatory cytokines which eventually involved in insulin resistance and dysfunction of *β* cell in type 2 diabetes [57, 63]. These studies suggest that ROS is increased in both types of diabetes.

ROS causes loss of insulin type 1 diabetes by destructing the *β* cells but it causes insulin resistance in type 2 diabetes by *β* cell dysfunction of [49, 55, 56]. Endothelium-dependent vasodilation is impaired in diabetic animals and humans. Excessive production of vascular superoxide contributes to this impairment of endothelium-dependent vasorelaxation [65]. There is evidence that pretreatment of diabetic rat aorta with SOD and/or antioxidant probucol prevents the impairment of endothelium-dependent relaxation in aortic rings [66]. Likewise, pretreatment with either SOD or catalase has been shown to improve endothelium dysfunction in streptozotocin-induced diabetic rats suggesting that vascular production of both superoxide and H_2_O_2_ may contribute to endothelial dysfunction [67]. Another relevant mechanism involves direct inactivation of endothelial-derived relaxing factor (EDRF) by advanced glycation end products, (AGEs) and increased adhesion of leukocytes to the endothelium [68]. Besides, this increased production of the superoxide anion can lead to decreased tissue bioavailability of nitric oxide (NO) via a facile radical/radical reaction that occurs more rapidly than the reaction of superoxide anion with superoxide dismutase [25]. This phenomenon alters endothelial regulation of vasomotion in a variety of disease conditions. Importantly, this endothelial dysfunction is due to vascular production of superoxide. Therefore, ROS-induced loss of insulin or insulin resistance is responsible for ultimate onset of type 1 and type 2 diabetes and their vascular complications, respectively. Finally, ROS-induced endothelial dysfunction is mediated by decreased NO bioavailability, inactivation of EDRF by AGEs that are common pathways in both type, of diabetes.

#### 4.1.7. Role of ROS in Impaired Angiogenesis in Diabetes

Cardiovascular complications are the leading cause of morbidity and mortality in patients with diabetes mellitus. In particular, diabetes is associated with a poor outcome after vascular occlusion. This can be attributed in part to impaired neovascularization [69]. Three principal events, vasculogenesis, angiogenesis, and collateral growth, contribute to postnatal vessel growth, and each may be affected by diabetes. Indeed, there are a number of equally tenable hypotheses regarding the mechanisms underlying alterations in blood vessel growth in diabetes, including a reduction in vascular endothelial growth factor-A (VEGF-A) signaling, changes in inflammation-related pathways, and accumulation of advanced glycation end products [70–72]. Postnatal vasculogenesis can also be affected because the proangiogenic effects of bone marrow mononuclear cells (BM-MNCs) and endothelial progenitor cells (EPCs) are reduced in diabetic mice and patients with either type 1 or type 2 diabetes [73]. Evidence suggests that the effects of ROS on vascular function depend critically on the amount of ROS present. However, precise amounts and the species of ROS involved are not fully understood. Low levels of ROS (principally H_2_O_2_) under physiological conditions can act as intracellular secondary messengers modulating proangiogenic pathways such as VEGF-A signaling and postnatal vasculogenesis; conversely, higher levels of ROS can impair neovascularization [74, 75]. These ROS include superoxide, hydrogen peroxide, hydroxyl radical, lipid peroxides, and peroxynitrite, which are recognized to play major roles in vascular biology stimulating redox signaling [75, 76]. Each of these species derives from specific enzymatic or chemical reactions as discussed earlier.

 Seminal works in experimental models of types 1 and 2 diabetes, as well as in human patients, suggest that there is a strong link between ROS and diabetes [75, 77]. Several studies have been performed in diabetic patients, animals, and in high-glucose-treated endothelial cells that implicate NADPH oxidase as an important source of hyperglycemia-induced ROS formation [69, 78–80]. Ebrahimian et al. demonstrated that diabetes-induced overproduction of ROS impairs postischemic neovascularization [69]. This study also reported that blockade of oxidative stress in the setting of diabetes restores key pathways involved in angiogenesis, such as VEGF-A signaling and postnatal vasculogenesis. However, NADPH oxidase activity and ROS production mediate angiogenesis in both cultured cells and *in vivo* models of neovascularization [81]. Nishikawa et al. suggest that glucose-induced mitochondrial production of ROS stimulates several biochemical mechanisms involved in diabetic complications, including retinopathy [46]. Studies indicate that superoxide production by NADPH oxidase has a primary role in VEGF expression and vitreoretinal neovascularization in a mouse model for ischemic retinopathy [82]. Moreover, increased expression of NADPH subunit NOX2 correlates with increases in ROS and VEGF and breakdown of the blood-retinal barrier during diabetic retinopathy [83]. In addition, superoxide overproduction by NADPH oxidase likely reduces NO bioactivity by scavenging or through uncoupling of endothelial nitric oxide synthase and may also lead to the formation of other signaling species such as peroxynitrite [77, 84]. These alterations in NO signaling might contribute to modulation of postischemic neovascularization because NO is a well-known mediator of BM-MNC mobilization and differentiation, as well as basal neovascularization reaction [85]. However, previous studies present evidence that NADPH oxidase activity and expression are significantly increased in diabetic tissue [77, 81]. Evidence shows that blockade of NADPH oxidase activity or the scavenging of ROS restores postischemic neovascularization in diabetes. The subcellular distribution of NADH oxidase activity responsible for the effects of diabetic retinopathy is not clear. Though there is evidence that NADH oxidase is expressed in retinal epithelium and retinal pericytes, there is no clear information on the contribution to increased angiogenesis [86]. In addition, there are no studies available implicating the exact role of VEGF in macular edema and retinal angiogenesis. Hyperglycemia-induced overproduction of ROS also impairs EPC function leading to impairment of angiogenesis and vasculogenesis in diabetes [87, 88]. But EPC implications on ROS and angiogenesis under diabetes is still not clear and a subject of extensive study.

 It has been reported that diabetes-induced increases in ROS-mediated p38MAPK phosphorylation in BM-MNCs reduce BM-MNC differentiation into EPCs *in vitro* and impair their proangiogenic potential *in vivo *[73]. Similarly, diabetes has been shown to activate p38MAPK in vascular cells via PKC-dependent and -independent pathways [89]. Moreover, p38MAPK activation is known to downregulate EPC proliferation and differentiation that may contribute to impaired vasculogenesis in diabetes. Antioxidant defense capacity is reduced in animal models of diabetes, and this can also contribute to diabetes-induced oxidative stress [90]. Chronic treatment of diabetic animals with N-acetyl cysteine (NAC) improved or normalized endothelium-dependent responses which may normalize impaired angiogenesis in diabetes [91]. Vasodilation, a primary initiation factor for arteriogenesis, is attenuated through ROS reduction of NO bioavailability during diabetes. There is strong evidence that collateral formation is impaired in diabetes [92]. This further substantiates that all three components of diabetic angiogenesis are impaired by ROS, whatever the source of production.

### 4.2. Decreased NO Production and EC Dysfunction

The vascular endothelium comprises the internal lining of blood vessels, which serves as an interface between the blood and smooth muscle cells. Endothelium is a key determinant of vascular health in addition to being a barrier between luminal contents and the vessel. Endothelial dysfunction, which is often related to impaired endothelium-dependent NO-mediated relaxation, occurs in both cellular and experimental models of diabetes [93–95]. Similarly, the majority of clinical studies have shown an abnormality in endothelium dependent vasodilation in patients with diabetes [96–98]. Thus, decreased levels of NO may underlie the atherogenic predisposition of diabetes. Many of the metabolic conditions associated with diabetes, including hyperglycemia, excess free fatty acid liberation, and insulin resistance mediate abnormalities in endothelial cell function by affecting the synthesis or degradation of NO [99].

 Endothelial dysfunction associated with insulin resistance appears to precede the development of overt hyperglycemia in patients with type 2 diabetes mellitus [100, 101]. Oxidative stress and insulin resistance have a direct relationship mediating diabetic cardiovascular complications. Insulin plays a critical role in the maintenance of physiological endothelial function through its ability to stimulate NO release via a cascade that involves activation of the PI3K-Akt signaling and downstream serine phosphorylation of eNOS. A key characteristic of insulin resistance is decreased PI3K signaling, increased mitogen-activated protein kinase (MAPK) activity, and increased secretion of ET-1, a consequence of endothelial dysfunction [102]. 

**Figure 3:**
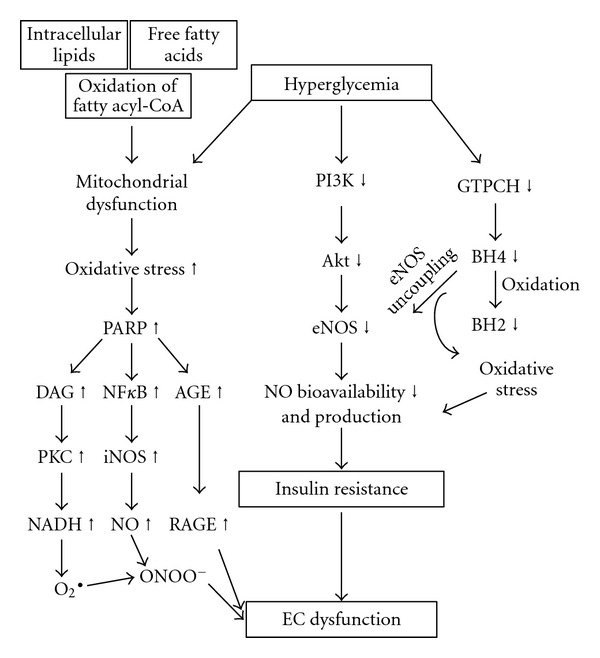
Signaling mechanisms leading to endothelial dysfunction under diabetes. Diabetes-mediated hyperglycemia leads to multiple-signal pathway dysfunction within vascular endothelial cells. Primary insults include mitochondrial dysfunction, defective PI3 kinase signaling, decreased NO production, increased oxidative stress, and differential PKC isoform activation. Key words: pARP: poly (ADP-ribose) polymerase; AGE: advance glycation end products; DAG: diacylglycerol; NF-*κ*B: nuclear factor kappa-B; PKC: protein kinase C; iNOS: inducible nitric oxide synthase; NADH: nicotinamide adenine dinucleotide; NO: nitric oxide; RAGE: receptor for advanced glycation endproducts; O_2_^∙^: superoxide anion; ONOO^−^: peroxynitrite; PI3K: phosphatidylinositol 3-kinases; AKT: protein kinase B; eNOS: endothelial nitric oxide synthase; GTPCH: GTP cyclohydrolase; BH4: tetrahydrobiopterin; BH2: dihydrobiopterin.

The majority of deaths occurring in patients with diabetes is due to vascular dysfunction. Studies have shown that endothelial dysfunction, as represented by impaired endothelium-dependent NO-mediated relaxation, occurs in diabetes [98, 103]. The first evidence of endothelial dysfunction in humans was reported in penile corpora cavernosa of IDDM and NIDDM patients [104]. High concentrations of glucose have been associated with endothelial dysfunction *in vivo* and *in vitro* [95, 105]. Mechanisms underlying this endothelial dysfunction could include decreased activity and/or expression of eNOS or increased degradation of NO secondary to enhanced superoxide production. More recent data support the concept of NO degradation, because treatment of vessels from diabetic animals with SOD improved endothelial-dependent relaxation and the use of vitamin C (another known antioxidant) in patients with non-insulin-dependent diabetes markedly increased endothelial-dependent relaxation in forearm arterioles [105, 106]. Mechanisms involved in decreased NO bioavailability and endothelial dysfunction under diabetes is depicted in Figure 3. Posttranslational modification of eNOS through the hexosamine pathway, downregulation of eNOS expressions and S-nitrosylation of eNOS have been the major causes for diabetic endothelial dysfunction [107–109].

#### 4.2.1. Causes of Reduction of NO Production Leading to ECD

**(1) Reduction of NO Bioavailability**

Nitric oxide is a key signaling molecule produced by vascular endothelial cells, which plays a vital role in the maintenance of vascular tone and other physiological processes of the cell. Cellular exposure to high glucose as seen in diabetes induces generation of reactive oxygen species (ROS) [110]. Another major abnormality that is commonly prevalent with diabetes is decreased in NO bioavailability [111]. A number of studies suggest that decreased NO bioactivity associated under hyperglycemia and diabetes is due to either quenching of normally released NO or impairment of NOS activity [100]. Reduced vascular production of NO is associated with uncoupling of eNOS due to ROS, reduced cofactors of eNOS such as L-arginine and tetrahydrobiopterin (BH4), and improper activity of BH4 producing enzyme GTP cyclohydrolase I [112, 113].

**(2) eNOS Phosphorylation**

Defects in Akt/eNOS signaling may play a primary role in endothelial dysfunction in type 2 diabetes mellitus. Studies have shown that Akt/eNOS phosphorylation is decreased in aortas of diabetic animals, as well as type 2 diabetic patients [114]. Activation of PI3K-Akt pathway eNOS-derived NO results in improved endothelial function and rescue of impaired myocardial cells [115]. Recent studies on eNOS gene disruption studies in mice revealed that deficiency leads to insulin resistance resulting in hypertension and hyperlipidemia [116]. Further biochemical studies in insulin-responsive cells have revealed a phosphorylation-dependent signaling role in insulin stimulated activation of eNOS [117]. Chen and Stinnett have shown in studies with diabetic mice that high glucose upregulated Ang-2 and downregulated Tie-2 expression leading to significant impairment of Ang-1-induced Akt and eNOS phosphorylation, which resulted in impairment of endothelial cell migration and sprouting [118]. Ang-1 gene transfer restored Tie-2 expression and rescued these abnormalities in diabetes.

O-GlcNAcylation protein modifications result in several diabetic complications. Studies show that O-GlcNAcylation of eNOS in endothelial cells is involved in micro- and macrovascular complications [119]. In a diabetic rat model, Musicki et al. showed *O*-GlcNAcylation modifications caused eNOS dysfunction in the penis thereby affecting phosphorylation of eNOS at the Ser1177 residue, which contributed to erectile dysfunction and long-term penile health issues for patients with diabetes [120]. In another study, Cho et al. showed in a diabetic mouse model that impairment of erectile function was caused by upregulation of expression of Rho-kinase 2 (ROCK2) and myosin phosphatase targeting subunit 1 (MYPT1) and decreased eNOS phosphorylation. Lima et al. have shown that elevated O-GlcNAc levels contribute to end-organ damage and vasoconstriction under diabetes, concurrent with decreased eNOS (Ser1177) and Akt phosphorylation (Ser473) [121]. These studies indicate that targeting abnormal O-GlcNAcylation, that is, associated with diabetes can enhance eNOS phosphorylation and thereby restore vasoregulation.

Taguchi et al. demonstrated in a streptozotocin-induced murine diabetic model that GRK2 is upregulated under diabetic conditions impairing Akt/eNOS activity by inhibiting their phosphorylation. However, phosphorylation of Akt at Thr308 was normalized and the phosphorylation of eNOS at Ser1177 was increased by GRK2-inhibitor [122]. Decreased phosphorylation of both Thr495 and Ser1177 residues in livers of diabetic mice was observed by Elrod et al., although there was no difference in total hepatic eNOS protein [123]. Sasso et al. demonstrated that in the hearts of diabetic patients with chronic coronary heart disease (CHD), there was downregulation of VEGF-dependent intracellular signaling and eNOS phosphorylation [124]. They reported reduced VEGF receptor Flk-1 phosphorylation concomitant with decreased Akt phosphorylation and decreased eNOS protein phosphorylation and expression. Studies also show that there is an influence of posttranslational modifications leading to decreased eNOS activity under diabetes [107, 125].

NO-based therapies have been proven by numerous investigations in various animal models [126–128]. Calvert et al. have shown that in diabetic mice under hepatic and cardiac I/R, treatment with metformin-augmented AMPK activation and significantly increased eNOS phosphorylation at serine 1177 residue [129]. Another study in diabetic rats by Penumathsa et al. demonstrated that Niacin-bound Chromium (NBC) treatments mediate translocation of Glut-4 leading to dissociation of Cav-1/eNOS interaction followed by increased phosphorylation of AMPK, Akt, and eNOS [130]. Ahanchi et al. have demonstrated that NO exerts protection in a rat carotid artery balloon injury model of type 2 diabetic obese rats. Their results show that topical administration of NO not only prevented neointimal hyperplasia following artery injury, but also reduced ROS production and cell death and inhibited VSMC proliferation in these animals [128]. Together, these studies indicate that diabetes-mediated endothelial dysfunction potentially alters eNOS phosphorylation and thereby NO production. Increases in Akt/eNOS phosphorylation or inhibition of the factors involved in repressing eNOS would rectify the vascular complications during diabetes. Akt/eNOS phosphorylation serves important roles in rectifying vascular defects during the pathology of diabetes; however, further studies are needed to explore different sites involved in eNOS phosphorylation during diabetic complications that affect NO production and thereby endothelial dysfunction.

**(3) eNOS Uncoupling**

Endothelial NOS (eNOS) derived nitric oxide (NO) in endothelial cells regulates vascular tone and plays a key role in maintaining endothelial health. Evidence from eNOS knockout mice states that functional eNOS is critical for the maintenance of vascular health [131, 132]. Proper functioning of the endothelium is often linked to the production and bioavailability of NO and relative regulation of ROS. Endothelial dysfunction occurs when there is a reduced bioavailability of NO. This reduced NO bioavailability and endothelial dysfunction is observed under hyperglycemic conditions both *in vitro* and *in vivo* [95, 97]. Decreased endothelial-dependent arterial relaxation is observed during diabetes in animal models as well as in human subjects [133].

Endothelial NOS acts as an active enzyme complex producing NO in its “homodimer” state during physiological conditions, while the enzyme is inactive and unable to produce NO under pathological conditions. Even though a concomitant increase in eNOS levels is observed under pathological conditions [134, 135], this state may generate superoxide anions from the monomerized eNOS instead of NO, a condition called “eNOS uncoupling” [136]. Studies indicate that endothelial nitric oxide synthase (eNOS) function is impaired in diabetes as a result of reduced NO bioavailability and increased vascular generation of reactive oxygen species [13, 137, 138]. Endothelial NOS uncoupling and nitrosative stress have been observed during vascular abnormalities such as hypertension, atherosclerosis, and diabetes [136].

**(4) Tetrahydrobiopterin (BH4)**

Endothelial NOS must be in an active dimer state to produce NO. Regulation of the dimeric eNOS complex is important for proper functioning of eNOS. L-arginine and BH4 are two critical factors that maintain the dimeric state of eNOS allowing electron flow across the homodimer to generate NO from the ferrous-dioxygen complex [134, 135, 139]. BH4 is a metabolite that serves as a critical cofactor and inhibits superoxide generation from the heme group at the oxygenase domain of eNOS [112]. BH4 acts as a redox regulator of eNOS by promoting and stabilizing eNOS protein monomers into the active homodimeric form [140], which in turn maintains the healthy state of the endothelium. Under reduced L-arginine or BH4 levels, eNOS functions in an “uncoupled” state in which NADPH-derived electrons are added to molecular oxygen rather than L-arginine, generating more O_2_^−^ as a product. O_2_^−^ generated by eNOS has been implicated in a variety of experimental and clinical vascular disease states including diabetes, hypertension, and atherosclerosis [141].

eNOS uncoupling and endothelial dysfunction are apparent in experimental models of diabetes and in diabetic patients [77, 84] despite the fact that eNOS expression is actually increased. Hyperglycemia results in BH4 deficiency and eNOS dysfunction characterized by a decrease in NO with a concomitant increase in superoxide production [111, 142, 143]. *In vitro* studies have demonstrated that high glucose levels reduce NO activity and increase superoxide levels coupled with reduced eNOS dimerization in endothelial cells [144–146]. Hyperglycemia results in significant reductions in both total biopterins and BH4. BH4 bioavailability is postulated to be limiting in several vascular disease states including diabetes. Peroxynitrite, a potent oxidant, rapidly oxidizes BH4 to BH3, and subsequently to BH2 [143, 147], which may compete with L-arginine for eNOS, resulting in impaired eNOS bioactivity [139].

There are several studies that suggested the role of BH4 in eNOS homodimerization. A study on bovine eNOS expressed in *E. coli* suggested that BH4 influences the heme environment and stabilizes the eNOS protein [148]. In another study it was shown that exogenously added BH4 increased both eNOS activity and dimerization [149]. It is interesting to observe that eNOS protein expression levels increase in endothelial cells in response to high glucose [113, 146], which indicates the uncoupling of eNOS due to decreased BH4. Although studies indicate that BH4 facilitates electron transfer and maintains the dimerized state of the enzyme, the complete role of BH4 in eNOS regulation is currently unknown.

BH4 has proven to be an established therapeutic agent for hyperphenylalaninemia. Its potential has also been evaluated for therapeutic efficacy in the reversal of endothelial dysfunction. Studies indicate that BH4 therapeutic interventions to improve endothelial function have limited success in animal models of type 2 diabetes and in human studies [150–152]. Conversely, studies have shown that supplementation of this cofactor restores eNOS-mediated NO formation and endothelial function in hypertension, hypercholesterolemia, and diabetes [135, 139, 141]. Experimental and clinical evidence suggests that BH4 or L-arginine can act as a therapeutic agent to restore diabetes-induced endothelial dysfunction [138, 151, 153]. Pieper has shown that *in vitro* treatment of BH4 over the aortic rings of diabetic rats restores endothelial function [154]. Heitzer et al. have shown that the attenuated endothelium-dependent vasodilation in the forearm of diabetic patients was considerably improved by concomitant treatment with BH4, but not endothelium-independent vasodilation responses [151]. Lastly, Cai et al. showed in HAEC cultured in high glucose that BH4 restores the physiologically normal enzymatic activity of eNOS [155]. Together, these findings suggest that supplementation of BH4 may be useful to alleviate vascular complications through restoration of endothelial functions/eNOS activity in type 2 diabetes patients.

**(5) GTP Cyclohydrolase**

While BH4 is known to be an essential cofactor for activity of all NOS enzymes, its synthesis is also important for vascular health [156]. BH4-synthesizing enzyme GTP cyclohydrolase I (GCH) and BH2 reducing enzyme dihydrofolate reductase (DHFR) counteract the intracellular depletion of BH4. GCH is the first-rate limiting enzyme for BH4 de novo biosynthesis through GTP catalyses [157]. GCH is constitutively produced in endothelial cells and its activity is crucial for BH4 bioavailability and proper endothelial function. Studies show that genetic overexpression of GCH can prevent endothelial dysfunction in diabetes [142, 158, 159].

Previously, it was reported that insulin can augment GCH activities in ECs through a PI3K-dependent pathway and that insulin-induced vasodilation depends on BH4 biosynthesis [160–162]. However, these mechanisms may be impaired in the insulin-resistant state. Hyperphenylalaninemic (Hph-1) mice are mutants with partially reduced GCH activity. Studies in the Hph-1 mouse suggest that BH4 deficiency leads to hypertension, increased vascular oxidative stress, and reduced eNOS activity, which demonstrates that reduced BH4 levels lead to eNOS uncoupling in the absence of vascular disease [163]. From these studies it is clear that increased endothelial BH4 biosynthesis by transgenic GCH overexpression can alter eNOS uncoupling and can improve vascular health. Mitchell et al. have reported in a glucocorticoid-induced rat model of hypertension that GCH mRNA levels were reduced, and impaired endothelium-dependent relaxations could be restored by incubating vessels in sepiapterin (BH4 precursor). This suggests that reduced BH4 bioavailability is a cause of eNOS uncoupling and vascular dysfunction [164]. Other studies in the DOCA-salt hypertensive mouse also showed that decreased BH4 levels were related to reduced GCH activity [165]. Increased GCH activity through exogenous gene delivery or BH4 supplementation reversed BH4 deficiency and endothelial dysfunction by reducing superoxide levels.

Study of isolated aortic rings from diabetic rats showed that overexpression of GCH by gene transfer reverses diabetes-induced BH4 deficiency and restores NO bioavailability [159]. In another study Meininger et al. showed that GCH-I activity is markedly decreased in animal models of types 1 and 2 diabetes, which contributes to endothelial dysfunction [159, 166–168]. Alp et al. showed that an increase in endothelial BH4 levels, NO bioavailability, and reduced endothelial superoxide production were observed in a transgenic human GCH overexpressor mouse model of diabetes compared to diabetic wild-type mice [158]. These studies strongly indicate that the depletion of BH4, NO bioavailability, and increased endothelial superoxide production are interrelated in the diabetic condition. While prevention of decreased BH4 levels through overexpression of GCH restores vascular function, other mechanisms such as endothelial GCH phosphorylation have yet to be explored during diabetes and other vascular complications.

**(6) Arginase**

Vascular dysfunction is a major cause of morbidity and mortality in diabetic patients [169]. Reduced L-arginine availability has been implicated as a cause of vascular dysfunction in diabetes and other diseases. Arginase, which catalyzes L-arginine to urea and ornithine, competes directly with NOS for L-arginine. Increase in arginase activity leads to decreased cellular arginine levels and its availability for eNOS thereby decreasing NO production and generation of superoxide by eNOS [170, 171]. Enhanced arginase activity has been implicated in a number of vascular dysfunctional states including diabetic erectile dysfunction [172–175].

There are two mammalian variants of arginase, arginase I and II, which are differentially expressed in various tissues [173, 176, 177]. Arginase I is localized to the cytoplasm and prominently expressed in the liver, whereas arginase II is located in the mitochondria and expressed in the kidney. Arginase activity increases in the liver of diabetic rats [178, 179]. Reports from diabetic animals and patients demonstrate that arginase activity is increased under diabetic conditions [180, 181], while decreased insulin signaling is associated with diabetic insulin resistance. It is important to note that insulin represses gene expression of urea cycle enzymes, thus increased arginase activity may be linked with decreased insulin signaling which requires further study.

Increased arginase expression reduces NO synthesis in diabetes resulting in impairment of normal endothelial functions such as vascular remodeling responses [182]. This suggests important roles played by arginase leading to many of the vascular complications observed during diabetes [183]. Arginase I has been reported to be upregulated in porcine coronary microvessels, which consequently lead to diminished vasodilation [175]. In another study Zhang et al. showed in pigs with experimental hypertension that NO-mediated dilation of coronary arterioles is reduced due to increased arginase I activity, which leads to limited availability of L-arginine [173]. Romero et al. showed that in coronary arteries of diabetic rats that arginase I activity results in a diminished NO-mediated response contributing to vascular endothelial dysfunction, due to decreased availability of L-arginine for eNOS [184]. Recently Grönros et al. demonstrated that type 2 diabetic Goto-Kakizaki (GK) rats have increased arginase II expression, along with coronary artery microvascular dysfunction [185]. Importantly, microvascular function was normalized after arginase inhibition highlighting that arginase activity diverts arginine from NOS and that arginase inhibition increases NO bioavailability and coronary microvascular function in the GK type 2 diabetic rat.

Studies with patients indicate that diabetes is associated with an impaired vasodilator function of coronary arteries and vasospasm that indicates coronary endothelial dysfunction [186–188]. Recently Beleznai et al. demonstrated that in patients with diabetes, arginase I is upregulated in coronary arterioles, which interferes with NO-mediated vasomotor responses [189]. In this study, the authors found that the presence of *N* ^G^-hydroxy-l-arginine, a selective inhibitor of arginase, or application of L-arginine restored ACh-induced coronary dilation in patients with DM. Interestingly, in nondiabetic patients with other vascular abnormalities either arginase inhibition or L-arginine supplementation failed to show any change in vascular responses, indicating that arginase targets the ACh-mediated response. Further study is needed to ascertain the role of insulin and other pathological factors that are affected by increased arginase 1 expression in diabetes. These findings suggest that targeting arginase could be a useful treatment of diabetic endothelial dysfunction.

**(7) Peroxynitrite's Role in NO Bioavailability and Endothelial Dysfunction**

Diabetes has been shown to increase the vascular formation of the NO/superoxide reaction product peroxynitrite. There are several experimental and clinical studies available that demonstrate the formation of peroxynitrite in various tissues during diabetes, especially within the endothelium [190–194]. Moreover, there are various mechanisms that underlie the peroxynitrite-induced diabetic complications [195].

Increased levels of peroxynitrite under high glucose conditions reduce BH4 production and also reduce BH4 producing enzyme GCH expression, contributing to eNOS uncoupling [168, 196]. Specifically, peroxynitrite rapidly oxidizes the active BH4 to inactive dihydrobiopterin (BH2), leading to eNOS uncoupling [197]. In addition, peroxynitrite causes eNOS uncoupling through 26S proteasome-dependent degradation of GCH leading to the release of zinc from the zinc-thiolate cluster of eNOS, which presumably leads to the formation of disulfide bonds between monomers [146]. Oxidative loss of BH4 may mediate some of the observed effects of increased reactive oxygen species production on endothelial function in vascular disease states [143, 197].

Peroxynitrite targets various biomolecules, leading to cardiovascular dysfunction through multiple mechanisms [25]. One of these includes activation of the nuclear enzyme poly(ADP-ribose), polymerase (PARP-1), which is involved in the development of diabetic cardiovascular dysfunctions [192]. PARP-1 leads to the production of inflammatory mediators such as inducible nitric oxide synthase (iNOS), intercellular adhesion molecule-1 (ICAM-1), and major histocompatibility complex class II [198, 199]. NF-*κ*B is a key molecule in regulating expression changes of these proteins. Overproduction of peroxynitrite can increase iNOS through NF-*κ*B activation in endothelial cells [200]. Nagai et al. have also shown that peroxynitrite increases *N* ^*ε*^-(carboxymethyl)lysine (CML), a major antigenic advanced glycation end-product (AGE), which activates cell-signaling pathways such as NF-*κ*B to enhance the expression of vascular cell adhesion molecule-1 (VCAM) [201], which is involved in vascular inflammation. Thus evidence from various studies mentioned above suggests that peroxynitrite is a major mediator of vascular injury under diabetic conditions and indicates that effective neutralization of peroxynitrite formation can be beneficial in restoring NO bioavailability and vascular health.

**(8) Glutathionylation**

Protein S-glutathionylation forms by a direct oxidation of a protein and reduced glutathione (GSH), by a thiol-disulfide exchange between a protein Cys and oxidized glutathione (GSSG), and also with S-nitrosoglutathione [202]. This emerging pathway provides an additional mechanism to regulate intracellular redox state and the generation of reactive oxygen and nitrogen species. Recent studies indicate the importance of oxidants that directly impact the function of tissues by altering the structure of protein cysteinyl thiols. Multiple modes of protein-cysteine oxidation, such as *S*-thiolation, *S*-nitrosylation formation, and intra- and intermolecular protein disulfides are already known to play a prominent role in redox regulation [203, 204]. Several cellular signaling mechanisms have been reported to be modified resulting from protein glutathionylation involving defective insulin signaling resulting from diabetic conditions, which include NF*κ*B, RyR1, K^+^ and ATP channels, PKC, aldose reductase, mitochondrial complex I, and sarcoplasmic/endoplasmic reticulum Ca^2+^ ATPase (SERCA) [205–207]. It is interesting to note that alterations in these signals were reported in defective insulin secretion from *β* cells, insulin sensitization in peripheral tissues, and complication-related cell injury and tissue damage in diabetes [208]. Moreover, there is increasing evidence of functional changes resulting from these glutathionylation modifications in diabetes, with protein posttranslational modifications playing an important role in the maintenance and progression of disease pathogenesis [208]. S-glutathionylation of proteins is the primary mechanism of thiol redox signaling and therefore has significant impact on the pathogenesis of diabetes.

Increased formation of glutathionylated Hb HbSSG represents a change in the oxygen carrying capacity of hemoglobin and tissue-specific glutathionylation, which may lead to differential cellular responses. Niwa et al. demonstrated that there are increased levels of HbSSG observed in diabetic and hyperlipidemic patients [209]. Increased oxidative stress, lipid peroxidation, and glutathione depletion are commonly observed in diabetic subjects without microangiopathy [210]. Vita et al. have demonstrated in patients with coronary artery disease that the impaired endothelial NO bioactivity is reversed upon L-2-oxo-4thiazolidine carboxylate (OTC) delivery, an intracellular GSH inducer [211]. These studies emphasize the role played by GSH in regulating endothelial functions during disease states. S-glutathionylation of eNOS is a crucial switch providing redox regulation of cellular signaling, endothelial function, and vascular tone. Some eNOS S-glutathionylation can be increased under conditions like hypertension, with impaired vasodilation that is restored by thiol-specific reducing agents reversing this S-glutathionylation. Chen et al. have recently shown that cysteine residues Cys689 and Cys908 are critical for normal eNOS function, which can be glutathionylated to produce superoxide [125].

Glutathione in its oxidized form (GSSG) has been shown to regulate the activity of several purified enzymes including carbonic anhydrase III, protein kinase C (PKC), and human aldose reductase (AR) [212]. For years, inhibition of AR in diabetes has been a popular therapeutic approach. Cys298, an active site of AR for thiol modifications, is known to regulate substrate binding [213, 214]. S-glutathionylation of AR, specifically at Cys298, inhibits its activity under normal glucose concentrations [215, 216]. Likewise, inhibitors of AR have proved to be effective for therapeutic intervention in diabetes [213]. Glutaredoxin (Grx) has been reported to be increased in the diabetic heart and retina of rats [217], since AR is a regulatory target for Grx, Grx-dependent inhibition additionally may further enhance AR inhibition. Future therapeutics could be aimed at targeted inhibition of AR-mediated glucose signaling, without affecting aldehyde detoxification to prevent diabetes-associated inflammation and other vascular abnormalities.

Sarco/endoplasmic reticulum Ca^2+^-ATPase (SERCA) actively transport cytosolic Ca^2+^ into the sarcoplasmic reticulum, thus quenching cytoplasmic Ca^2+^ signals and regulating calcium oscillations in response to glucose [218]. Nitric oxide (NO) stimulates SERCA to decrease intracellular Ca^2+^ thereby allowing relaxation of cardiac, skeletal, and vascular smooth muscle [205]. High glucose has been reported to prevent NO-induced inhibition of VSMC migration due to Cys674 to serine mutation of SERCA, where this Cys is also subject to sulfonic acid formation in VSMC resulting in glutathionylation [219]. These results suggest a scenario involving increased oxidized thiol resulting in deglutathionylation modification of SERCA-Cys674-SH to the sulfonic acid which leads to protein glutathionylation affecting protein function.

Insulin and downstream signaling critically regulate NO and associated endothelial cell functions [220]. Studies provide evidence that glutathionylation of Cys118 and activation of Ras lead to endothelial insulin resistance, which was only recovered with Grx overexpression, implicating a role for Grx as a target treatment in diabetes [221]. Decreased Akt activity due to high glucose has been reported in diabetic rats and endothelial cells [222]. Changes in Akt activity are implicated in multiple signaling cascades that can be regulated by glutathionylation or interaction with Grx [223]. The mechanism of regulation of Akt phosphorylation by Grx is still not resolved. However, Murata's group proposed that the GSH/Grx system can protect Akt oxidation induced by H_2_O_2_ through glutathionylation of Akt cysteine residues Cys-297 and Cys-311 [224]. Wang et al. considered that this Akt protection is via deglutathionylation of upstream activators such as protein kinase A (PKA) [223]. Protein kinase C (PKC) is a major pathway that has tissue-specific implications under diabetic and vascular complications [225]. PKC isozymes can be oxidatively inactivated by S-glutathiolation involving endogenous thiols such as GSH [226]. Clinical trials have shown that Ruboxistaurin, a PKC-*β* inhibitor, can induce vascular protection of diabetic retinopathy [227]; however, Grx-mediated deglutathionylation of PKC may prove to be an additional therapeutic target for diabetic vascular complications.

NO synthesis is impaired in glutathione- (GSH-) depleted endothelial cells and GSH is reduced in patients with type 2 diabetes mellitus (T2DM) [228]. Martina et al. have shown that administration of GSH in patients with T2DM is able to improve platelet constitutive NOS (cNOS) activity together with a reduction of plasminogen activator inhibitor (PAI-1) [229]. Endothelial cell NO bioactivity is relatively sensitive to manipulations of intracellular GSH [230]. Studies have shown that thiol-manipulating agents altered endothelial NO bioactivity through mechanisms independent of changes in intracellular GSH [211]. In particular, protein thiol oxidation with diamide appears to have important implications for endothelial cell NO bioactivity by a direct effect on eNOS catalytic activity [231]. Further studies have to be performed to understand different changes in thiol residues that regulate endothelial NO activity under diabetic condition.

#### 4.2.2. Role of Endothelial Progenitor Cells in Endothelial Dysfunction and Diabetes

Endothelial progenitor cells (EPCs) are critical for maintenance and repair of endothelial cells. They play an important role in angiogenesis as they proliferate, migrate and differentiate, and are a source for proangiogenic cytokines [232]. EPCs express markers of both hematopoietic stem cells (CD34 and CD133) and endothelial cells (CD146, vWF, and VEGFR2) [233–235]. EPC dysfunction could contribute to the pathogenesis of vascular disease. There are numerous studies that have demonstrated, in patients with diabetes and cardiovascular disease, that the number of EPCs from peripheral blood is reduced and EPC function impaired [73, 88, 236, 237].

 Reports suggest that the number of circulating EPCs is decreased under both types 1 and 2 diabetes, which is likely to be involved in the pathogenesis of vascular complications [88, 238, 239]. In diabetes, the bone marrow derived EPCs are dysfunctional, producing fewer endothelial cells with reduced proliferative, and migratory potential due to oxidative stress [88]. EPCs act as a surrogate marker of vascular health and indicate cardiovascular risk in healthy persons [87, 240, 241]. In diabetic patients with vascular complications, there is a marked reduction of circulating EPCs compared to those patients without vasculopathy, and EPC counts correlate with the severity of vascular disease [87].

 Studies performed *in vitro* show EPCs from diabetic populations result in endothelial cells with a reduced capacity to form tubes, thereby inhibiting their ability to revascularize damage tissues [239, 242]. Kielczewski et al. demonstrated in renal occlusion model of C57BL/6J.gfp chimeric mice that insulin-like growth factor binding protein- (IGFBP-) 3 modulates vascular development by regulating EPC migration and restores the function of injured vasculature and NO generation [243]. In another study, Feng et al. showed in umbilical cord-derived EPCs that oxidized low-density lipoprotein (OxLDL) inhibits EPC survival and impairs their function, which may lead to inhibition of eNOS [244]. Recently Reinhard and colleagues have reported that in patients with type 2 diabetes on multifactorial treatments designed to improve glycemic control, lower lipids, reduce hypertension, and thrombosis, there was a significant increase in the number of EPCs [245]. Vasa et al. showed that in patients with coronary artery disease the number and migratory activity of EPCs are reduced, which may contribute to impaired vascularization [236]. Sorrentino et al. have demonstrated that the reendothelialization capacity of EPCs derived from patients with diabetes is severely impaired due to oxidative stress and reduced NO bioavailability [246]. In another publication, Thum and coworkers have attributed this deficiency to eNOS uncoupling as a result of diminished tetrahydrobiopterin (BH4) levels caused by EPC dysfunction in diabetic patients [247]. However, further studies are required to verify that increasing EPC numbers will improve diabetic anomalies. EPCs have also been suggested to function as activators of mature ECs through secretion of angiogenic factors [248]. These studies provide evidence that EPCs play a crucial role in regulating eNOS and endothelial functions under vascular dysfunctions.

 It is known that under diabetic conditions there are increased oxidative stress levels [15]. Increased ROS prompts the EPCs to produce pathologic cytokines such as monocyte chemoattractant protein-1 (MCP-1), tumor necrosis factor-*α* (TNF-*α*), NF-*κ*B, interleukin-8 (IL-8), elevated levels of iNOS, and decreased eNOS. The reduced functional activity of EPCs during hyperglycemia involves the Akt/eNOS pathway, where signaling is downregulated under diabetic conditions [249]. Ii et al. have attributed the phenotypic differences of EPCs during diabetes to decreased thrombospondin-1 expression [250]. There is an indication that upregulation of cyclin-dependent Kinase (CDK) inhibitors p16 and p21 leads to a reduction in proliferating EPCs under hyperglycemic conditions [251]. Information on molecular mechanisms influencing EPC numbers under diabetic or vascular dysfunctions is still sparse and deserves further research to better understand molecular mechanisms responsible for EPC formation and function.

 Therapeutic strategies could take advantage of EPCs ability to deliver cytokines and growth factors to diseased tissue to induce revascularization. Identifying the key modulators of physiologically normal functioning EPCs is essential in determining potential targets for restoring proper EPC function in diabetic populations. Clinical trials by Hamano et al. have shown that therapeutic angiogenesis induced by local implantation of autologous bone marrow cells led to recovery in patients with ischemic heart disease [252]. Studies performed by Strauer et al. showed similar effects, thus providing further evidence of the therapeutic potential of EPCs [253]. Recently, Wang et al. demonstrated that EPC dysfunction in diabetes may be caused by decreased manganese superoxide dismutase (MnSOD) expression [254]. In their study they also stated that in diabetic EPCs, expression of protein phosphatase 2A (which inactivates AMPK) was upregulated. Systemic hyperoxia is an adjunctive therapy to stimulate wound healing in diabetic patients, approved by the United States Food and Drug Administration (FDA). Previous studies showed that hyperoxia increases NO levels in vascular tissues via NOS stimulation [255], and bone-marrow-derived NO increased the number of circulating EPCs in nondiabetic models [256]. In a study focused on improving the number of circulating EPCs in a model of diabetes, Gallagher et al. showed that hyperoxia reversed the diabetic defect in EPC mobilization, which is a NO-mediated effect [257]. Stromal cell-derived factor-1*α* (SDF-1*α*), a chemokine that increases EC migration and angiogenesis mediated through NO [258, 259]. SDF-1*α* mediates EPC recruitment in ischemia, reversed the diabetic defect in EPC homing [257]. Desouza et al. have published recently that reduced activity and survival of EPCs in diabetic rats are caused by elevated NF-*κ*B levels, which results in decreased phosphorylation of Akt. This can be ameliorated by knockdown of NF-*κ*B, which restores insulin signaling, improves EPC survival, and decreases neointimal hyperplasia [260].

 Numerous studies demonstrated the positive effects of EPCs in repair processes of wound healing, ischemic repair, limb ischemia, endogenous endothelial repair, and neovascularization [261–263]. On the contrary, EPCs contribute to pathological neovascularization, and recent studies show that circulating EPCs are reduced in patients with nonproliferative diabetic retinopathy (NPDR) but increased in patients with proliferative diabetic retinopathy (PDR) [264–266]. These findings suggest the flipside of the EPCs that they may be associated with proinflammatory and proangiogenic EPCs, which lead to pathological neovascularization as observed in PDR. Studies on diabetic retinopathy lead to explore the possible role of EPCs in tumor angiogenesis [267]. Lyden et al. have demonstrated in angiogenic defective tumor resistant Id-mutant mice model that tumor angiogenesis is associated with circulating EPCs [268]. Their results state that impaired VEGF-driven EPC proliferation causes defective angiogenesis in this mice model. EPCs induce the endothelial cells leading to neovascular formation followed by cytokine-mediated recruitment of pro-angiogenic mural cells at the site of tumor growth [269, 270]. However, the brighter side of EPCs as a therapeutic modality is more, compared to its caveats. The promising results coming from research with EPCs warrant future studies into therapeutic uses of EPCs for treatment of vascular disease in diabetic populations.

### 4.3. Decreased Growth Factors and Cytokines in DM Results in Impaired Angiogenesis

Expression of various angiogenic growth factors is reduced during diabetic ischemia. Rivard et al. have shown that both VEGF protein and mRNA levels are decreased in ischemic muscles of type 1 diabetic mice [70]. The authors also showed that VEGF therapy restored blood flow in nonobese diabetic (NOD) mice. Insulin resistance also causes decreased expression of VEGF in type 2 diabetes [271]. Nitric oxide also plays a role in the angiogenic action of growth factors such as VEGF, FGF, and TGF-*β*. The induction of angiogenesis by these growth factors is blocked by NOS inhibitors [272, 273]. Collateral formation is impaired in diabetic patients and animal models of diabetes [70, 92]. Monocytes/macrophages are the major players in collateral formation. Waltenberger and colleagues have shown that VEGF-dependent monocyte function is severely impaired in diabetic patients [72]. Hyperglycemia and increased AGEs in diabetes cause defective VEGF signaling including inactivation of the VEGF receptor, FLK-1, which affects endothelial growth and migration, monocyte, and EPC recruitment and release from bone marrow. These defects also contribute to impaired arteriogenesis in diabetic ischemia [274]. EPC release from bone marrow, recruitment and homing to the ischemic site, is important for postnatal vasculogenesis, which is defective in diabetes. VEGF and SDF-1*α*, which promote EPC recruitment to the ischemic site, are impaired during vasculogenesis in diabetes [257]. FGF levels are also decreased in skeletal muscle, which impairs angiogenesis during diabetes [275]. Angiopoetin and its receptor, Tie 2, also play roles in impaired angiogenesis in diabetes [276]. Tanii et al. have suggested that PDGF-BB is decreased in STZ-induced type 1 diabetic mouse hind limb ischemia [277]. All of the above findings indicate that defective growth factor expression and signaling during diabetes impairs all three processes of neovascularization in diabetes.

### 4.4. Immune Cell Dysfunction in Diabetes Causing Defective Peripheral Angiogenesis

Reduced chemotaxis has been reported in polymorphonuclear neutrophils (PMNs) of diabetic patients (type 1 and type 2) than in those of healthy subjects [278, 279]. Another study corroborated reduced leukocyte chemotaxis in patients with hyperglycemia [278]. Since most PMN functions are energy-dependent processes, an adequate energy production is necessary for an optimal PMN function [280]. Glucose needs insulin to stimulate uptake into PMNs to generate this energy, which may explain the improvement of the chemotactic response after the addition of these two substances [281]. There is conflicting information regarding adhesion of PMNs in DM patients as some have shown decreased adhesion and others have shown no alteration [278]. Impairment of phagocytosis is found in PMNs isolated from poorly regulated patients. Cytokine release is decreased after stimulation of PMN in diabetes [278, 282]. Impaired chemotaxis and phagocytotic properties of monocytes are observed in diabetic patients. Plasma from healthy control subjects or addition of insulin does not cause any significant change in the phagocytotic capacity of diabetic monocytes, it seems that this impaired function is caused by an intrinsic defect in the monocytes themselves [283]. In addition to the decreased production of proinflammatory cytokines following LPS stimulation, monocyte/macrophage functions are also impaired in DM type 1 patients. The cellular response of monocytes to VEGF-A is attenuated in diabetic patients [72]. Impaired chemotaxis and monocyte phagocytotic activity leads to reduced cellular innate immunity, thereby increasing the prevalence of infections and decreasing growth factors which impair wound healing and angiogenesis in DM patients.

### 4.5. Differences in Impaired Angiogenesis in Type 1 and Type 2 Diabetes after Ischemia

Types 1 and 2 diabetes differ in disease onset, pathophysiological mechanisms, and symptom severity. Likewise, restoration of blood flow after ischemia in both types of diabetes also differs. An interesting study by Yan et al. has shown that blood flow recovery was delayed and less effective in type 2 diabetes compared to that in type 1 diabetes [284]. Results from this study identified that capillary/myofiber ratio and arteriolar size were more severely diminished in type 2 diabetes due to attenuated eNOS expression in ischemic tissue and EPCs. Oxidative stress, as observed through nitrotyrosine formation, was preferentially increased in ischemic tissue in type 2 diabetes [284]. EPC migration and incorporation of EPCs into tubular structures was less effective in type 2 diabetes. The tubule formation defect in EPCs may explain the difference in impaired angiogenesis and arteriogenesis following chronic ischemia in experimental type 2 diabetes. Rivard et al. have reported that exogenous VEGF rescues impaired blood flow in type 1 diabetic NOD mice [70]; however, some authors suggest that growth factor or gene therapy may be insufficient as a sole strategy to enhance type 2 diabetic revascularization [274]. This result again indicates the severity of impaired neovascularization in type 2 diabetes. One of the important sources of conflicting findings in diabetic angiogenesis may be the use of diverse animal models to induce diabetes, the models themselves, and the effect of ischemia on angiogenesis in these models.

#### 4.5.1. Diabetic Wound Healing

Wound healing occurs as a cellular response to injury and involves activation of keratinocytes, fibroblasts, endothelial cells, macrophages, and platelets. These cell types coordinate and maintain healing through the release of many growth factors and cytokines. Defective immune cell responses or impaired recruitment within the wound site results in defective healing in diabetes. Prolonged diabetes leads to impaired wound healing, a result of defective angiogenesis [72, 92]. Foot wounds followed by ulceration are a leading cause of hospital admissions for people with diabetes throughout the world and is a major comorbidity associated with diabetes, leading to extreme pain and suffering and poor quality of life for patients. Data have shown that diabetic foot ulcers (DFUs) are estimated to occur in 15% of all patients with diabetes [285] and precede 84% of all diabetes-related lower-leg amputations [286]. There are several ways that uncontrolled diabetes can lead to diminished wound healing. Firstly, diabetic individuals often are unable to combat infection due to defective immune responses. Thus, even small scrapes can transition to open, infected sores. Secondly, nerve damage in diabetic patients' results in lack of peripheral sensory function. Nerve damage may be prominent in diabetic patients resulting in a diminished capacity to notice cuts, blisters, or ulcers. Thirdly, diabetic individuals typically have diffuse atherosclerotic vessel disease that diminishes blood perfusion leading to a disruption in wound oxygenation and healing [287]. Lastly, the DFU may also become a portal for systemic infection leading to bacteremia, septicemia, and may result in limb amputation. Importantly, delayed healing of diabetic wounds is also characterized by impaired angiogenesis and vasculogenesis responses [20]. A series of multiple mechanisms, including decreased cell and growth factor response, lead to diminished peripheral blood flow and decreased endothelial cell proliferation and contribute to the lack of wound healing in diabetes. Excessive ROS production in diabetic patients is a primary factor contributing to wound healing deficiencies, which can be reversed using ROS antagonists [288]. Decreased or impaired production of NO in DM is mainly due to impairment of eNOS phosphorylation and deficiency of arginase. There is evidence that NO produced during the healing process clearly regulates and augments wound repair [289]. Frank et al. reported that wound healing and angiogenesis are impaired due to reduced eNOS- and iNOS-dependent NO production which could also affect growth factor expression [290]. A recent study showed that increased ROS delayed wound healing and treatment with eNOS and MnSOD rectified poor diabetic wound healing. Antioxidants such as vitamin E have also been reported to accelerate diabetic wound healing, angiogenic responses, macrophage function, collagen accumulation, epidermal barrier function, granulation tissue formation, keratinocyte and fibroblast migration and proliferation, number of epidermal nerves, bone healing, accumulation of extracellular matrix (ECM) components, and their remodeling through matrix metalloproteinase (MMPs) [291–293]. Imputed defense responses like defective phagocytic granulocyte function and decreased granulocyte chemotaxis lead to impaired wound repair in diabetic patients [294]. Nolan et al. suggested that diabetic ulcers are more prone to impaired granulocytic function and chemotaxis [295]. Fang et al. suggested that GM-CSF is reduced in diabetic wounds and treatment with exogenous GM-CSF enhances wound healing in diabetes [296]. Nonetheless, prolonged inflammation, impaired neovascularization, decreased extracellular matrix remodeling, increased levels of proteinases, and defective macrophage activity all contribute to poor wound healing in diabetes.

 Bone-marrow-derived EPCs may also play a significant role in the healing of diabetic wounds. Gallagher and colleagues reported that EPCs in the bone marrow respond to chemokine gradients of VEGF and SDF-1*α*, which result in the homing of these cells to sites of hypoxia where they then participate in the formation of new blood vessels [257]. Bone-marrow-derived EPCs are mobilized to wound sites by eNOS activation in the bone marrow which is impaired in diabetics [257]. EPC recruitment to the wound site depends on upregulation of SDF-1*α*. Gallagher et al. also reported a decrease in SDF-1*α* expression particularly by epithelial cells and myofibroblasts derived from wounds of streptozocin-induced diabetic mice; this decrease was responsible for decreased EPC homing [257]. There is evidence that expression of growth factors apart from VEGF, such as FGF or PDGF-BB, is also implicated in decreased diabetic wound healing. Fibroblast delivery of PDGF-BB through an absorbable mesh is a clinically efficacious drug therapy approved by the FDA [297, 298]. Thus it is possible that simultaneous combined therapies such as upregulation of growth factors and potential treatments targeting eNOS activation and EPC recruitment might secure better healing in diabetes.

### 4.6. Diabetic Ocular Dysfunctions

There are several possible mechanisms of excessive angiogenesis in diabetes such as hypoxia, upregulation of growth factors, integrins, oxidative stress, AGEs and fibronectins, and others [299]. Among the growth factors, VEGF has been shown to have potent proangiogenic activity both *in vitro* and *in vivo*. VEGF is an EC-specific mitogen, a chemotactic agent for EC and monocytes [72]. VEGF can also recruit EPC to ischemic sites [8, 300]. There is also evidence that human recombinant VEGF induces pathological vascular symptoms similar to diabetic retinopathy in nonhuman primates. Williams suggested that VEGF can be induced and stabilized by hypoxia, hyperglycemia, and various cytokines such as TGF-*β* and IL-1 [301]. It has been reported in patients of diabetic retinopathy that there are abnormal levels of VEGF in vitreous and aqueous humor. Increased levels of VEGF and FGF at the site of abnormal angiogenesis were also reported in patients with diabetic retinopathy and nephropathy, respectively [302, 303].

 VEGF expression is elevated in diabetic retinopathy by increased ROS levels mediated through AGEs [304]. H_2_O_2_ stimulates cell migration and proliferation in endothelial cells, and ROS directly modulates VEGF-A expression and vascular smooth muscle cell proliferation [304]. It has also been suggested that both the gp91^phox^-containing NADPH oxidase and Rac1 play a major role in VEGF-A-induced endothelial cell proliferation [69]. These studies indicate that AGEs induce angiogenesis through differential signaling under diabetic retinopathy, through ROS generation.

 Recently, possible involvement of inflammation in diabetic retinopathy has been recognized. Proinflammatory cytokine like TNF-*α* is identified as an initiator of inflammatory reactions in retinas of patients with diabetic retinopathy and rodent model of diabetes mellitus [305, 306]. Increased expression of inflammatory mediators such as IL-1*β*, CCL5, and CXCL12, and adhesion molecules such as ICAM-1 and VCAM-1 in diabetic retinopathy patients also increase inflammation in the vessels [307–309]. In addition, leukocytes recruitment at the vascular endothelium is a factor of inflammation in diabetic retinopathy [310]. Mcleaod et al. reported that numbers of neutrophils are significantly elevated in both retinal and choroidal vessels from diabetic patients that correlate with upregulation of ICAM-1 and P-selectin in the vessels [311]. These studies comprehensively suggest that diabetic retinopathy may also be an inflammatory disease.

 Integrin adhesion molecules are necessary for cellular migration and organization of growth factor signaling within extracellular compartment to induce angiogenesis [312]. Studies reveal that many endothelial cell integrins such as *α*1*β*1, *α*2*β*1, *α*4*β*1, *α*5*β*1, *α*6*β*1, *α*6*β*4, *α*9*β*1, *α*v*β*3, and *α*v*β*5 are involved in the regulation of endothelial functions leading to angiogenesis [313]. Casaroli Marano and his colleagues reported that integrin *α*5*β*1 is upregulated in diabetic retinopathy [314]. Moreover, blockage of integrins leads to blunt the motility and growth of cells necessary for angiogenesis in hypoxia-induced retinal neovascularization [315]. Hyperglycemia causes overexpression of fibronectin that in turn degrades into a proangiogenic form of fragmented fibronectin that results in aberrant angiogenesis, as observed in diabetic retinopathy [316]. These studies indicate that in patients with diabetes integrins induce proangiogenic signaling resulting in aberrant signaling under diabetes that is characteristic of diabetic retinopathy, nephropathy, and macrosomia.

### 4.7. Endothelial Cell Dysfunction—Diabetic Therapy

Diabetes is a metabolic disorder characterized by impaired endogenous insulin secretion and activity, reduced NO production and increased production of free radicals, or impaired antioxidant defenses. The predominant factor in diabetes-mediated complications is endothelial dysfunction. The mechanisms that lead to endothelial dysfunction in diabetes are complex. Single therapy may not adequately improve endothelial function, so it is necessary to target multiple factors for therapeutic intervention of endothelial dysfunction. There are numerous risk factors that can cause endothelial cell damage under diabetes such as hyperglycaemia, insulin resistance, dyslipidaemia, increased oxidative stress, inflammation, and hypertension [317, 318]. Most interventions targeting more than one risk factor of endothelial damage only can improve endothelial functions [319]. Treatments that improve endothelial function systemically, like ACE inhibitors, statins, metformin, antioxidants, folate, PKC-inhibitors, and supplements like L-arginine, BH4, folic acid, and polyphenols also appear to provide protection from diabetes mediated vascular events [320–324]. There are several clinical trials investigating the therapeutic regulation of endothelial function in patients with type 2 diabetes mellitus [320–322, 325–328]. However, there is no single therapy to date that can provide complete protection from diabetes-induced vascular events.

#### 4.7.1. Antioxidant Therapy

Increased free radical generation represents vascular endothelial dysfunction in type 1 and type 2 diabetes [9]. Antioxidant therapy has been an easy and well-known choice to reduce diabetes-mediated vascular abnormalities. Previous studies showed that there is an improved endothelium-dependent relaxant response with various antioxidant agents, including superoxide dismutase (SOD) [329, 330]. This paradigm has gradually shifted as further studies demonstrated that antioxidant therapy alone is not sufficient; results with various antioxidants, namely, vitamins E and C, have had disappointing results [331, 332]. Now it is almost certain that antioxidant therapy is an option that must be used in combination with other therapies to alleviate vascular abnormalities.

 In a study Ting et al. demonstrated that intra-arterial administration of vitamin C (24 mg/min) in diabetic subjects, augmented methacholine mediated endothelium-dependent vasodilation, whereas this is not reflected in nondiabetic subjects [105]. In another study by Timimi et al. in insulin-dependent diabetes mellitus patients, vitamin C selectively restored the impaired endothelium-dependent vasodilation in the forearm resistance vessels of these patients. These findings indicate that adequate scavenging of oxidant radicals by parenteral administration of ascorbate (vitamin C) restores endothelium-dependent vasodilation in both type 1 and type 2 diabetes [105, 333].

 Koo et al. have shown in diabetic rats that antioxidant therapy was ineffective when administered alone and was effective only when combined with insulin treatment [334]. Results of their work show that insulin therapy results in significant, but incomplete reduction in blood pressure and other ROS-mediated parameters, while antioxidant therapy alone had no effect on these parameters. However, combined insulin and antioxidant therapies show the desired effects in diabetic animals. Beckman et al. in their study on diabetic patients receiving oral vitamin C (1,000 mg) and vitamin E (800 IU) daily or matching placebo for 6 months showed that oral antioxidant therapy improves endothelium mediated vasodilation in type 1 but not type 2 diabetes [335]. There are certain clinical trials that state that vitamin E supplementation reduces cardiovascular events in individuals with diabetes mellitus and the Hp 2-2 (haptoglobin, a major antioxidant protein) genotype [336].

Although many pathways are invoved in ROS-induced endothelial dysfunction in both types of diabetes, few effective antioxidant approaches have achieved clinical success. Various factors make traditional antioxidant therapy inefficient at mediating oxidative stress during diabetes. Antioxidants such as vitamin E or C are required in extremely high concentrations to reduce levels of peroxynitrite. Moreover, there is insufficient evidence to demonstrate that vitamin E reaches target cells. Over the last decade several studies have suggested that antioxidant therapy only delays diabetes-induced endothelial dysfunctions, rather than providing complete recovery.

#### 4.7.2. Metformin

Metformin is a first-line oral antidiabetic drug of choice in the biguanide class of drugs. Metformin reduces LDL cholesterol and triglyceride levels and is the only antidiabetic drug that has been shown to prevent cardiovascular complications caused by diabetes [337, 338]. Metformin targets to ameliorate the insulin resistance mainly in the liver and muscle, thereby lowering blood glucose. Metformin primarily reduces the hepatic glucose output by regulating gluconeogenesis [337, 339, 340].

Previous studies by Mather et al. also showed that metformin improved vascular endothelial functions and insulin sensitivity in patients with type 2 diabetes [320]. De Jager et al. have shown that in patients with type 2 diabetes treated with insulin, metformin treatment was associated with improvement of endothelial function by decreasing expression of VCAM-1, E-selectin and PAI-1, which were not related to changes in glycemic control [341]. In another study Vitale et al. showed that metformin improves both insulin resistance and thereby endothelial function, measured by the homeostasis model, in patients with metabolic syndrome [342]. In a clinical study De Aguiar et al. demonstrated the endothelial protective effects of metformin in patients with diabetes and metabolic syndrome. In their study metformin leads to decreased weight, BMI, systolic blood pressure, and fasting plasma glucose, and improved lipid profile. Endothelium-dependent forearm blood flow (FBF) responses were also improved [343].

 In contrast to these reports there are studies that report metformin has no significant effects on endothelium in patients with type 2 diabetes [344]. The UK Prospective Diabetes Study showed that although monotherapy with metformin and also sulfonylureas or insulin can achieve good glycemic control initially, sustained control with these agents fails in 50% of patients after three years [345]. There should be multiple therapies which target different aspects of diabetic abnormalities to protect vascular function and obtain adequate long-term glycemic control. Metformin causes this beneficial effect through several mechanisms: (1) direct reduction of insulin resistance in type 2 diabetes, (2) antioxidant effects in both types of diabetes, which ultimately increases NO bioavailability, and (3) direct effect on vascular endothelial and smooth muscle cells causing vasorelaxation [320]. All of these will finally improve endothelial dysfunction in diabetes.

#### 4.7.3. AMPK an Emerging Therapy for Vascular Dysfunction in Diabetes Mellitus

Studies show that metformin activates AMP-activated protein kinase (AMPK), an enzyme that plays a key role in insulin signaling and glucose metabolism [346]. Metformin requires AMPK to induce its inhibitory effect on glucose production by liver cells [347]. This concept is further strengthened by the studies from Kim et al. who demonstrated that hepatic SHP gene expression induced by metformin requires AMPK and further inhibits the expression of hepatic gluconeogenic genes [338]. However, the AMPK-metformin crosstalk has not been well studied. Even pharmacological agents such as statins, thiazolidinediones, and rosiglitazone, to mention a few, are mediated in part by activation of AMPK in endothelial cells [348]. AMPK regulates eNOS activity and promotes eNOS association with heat shock protein 90 (HSP90) [349, 350]. Studies show that AMPK is involved in suppression of inflammatory agents such as NF-*κ*B, regulating ROS/ONOO^−^, and also inducing mitochondrial biogenesis via PGC-1*α* induction in the endothelium [349]. In another study, adiponectin exerted cardioprotective affects during myocardial ischaemia-reperfusion that involves AMPK activation and production of endothelial NO thereby improving endothelial functions [351]. These studies show that AMPK contributes to prevention of ischemic heart disease through eNOS bioactivity and endothelial function. Further studies are warranted to explore the role of AMPK as a therapeutic agent for diabetes.

#### 4.7.4. Dipyridamole

Dipyridamole is a well-known antiplatelet agent, which is used with aspirin for ischemic stroke treatment and to restrict the progression of arterial occlusive disease [352]. Dipyridamole may inhibit adenosine uptake and cGMP-specific phosphodiesterases (PDE), thereby potentiating cGMP-mediated nitric oxide actions [352, 353]. De La Cruz et al. showed that aspirin plus dipyridamole showed prevention of ischemic cerebrovascular events like inflammation, as compared with other antiplatelet drugs or aspirin alone [354]. Vallon and Osswald have shown in an early diabetes model of rats that daily treatment with dipyridamole rectified diabetic kidney function by reducing interstitial adenosine concentrations in the kidney [355]. Dipyridamole may also augment coronary collateral development and cardiac function after ischemia/reperfusion injury [356, 357].

Dipyridamole also induces neuroprotection, antiplatelet effects, prolonged angiogenic effects, and an antioxidant effect [356, 358–360]. According to this “radical theory,” ischemic tissue injury associated with ischemia-reperfusion determines an increased oxidative stress which can contributes significantly to worsening of tissue injury [361]. There are studies that show direct powerful antioxidant properties of dipyridamole which protects NO bioavailability [362, 363]. Recent studies by our group also showed that dipyridamole therapy stimulates arteriogenesis during chronic hind-limb ischemia involving an endocrine NO/nitrite system [364].

Iuliano et al. have demonstrated that dipyridamole exhibits an antioxidant effect in inhibiting lipid peroxidation of methyl linoleate, and in the oxidation of low-density lipoprotein (LDL) [365]. Kusmic et al. observed that dipyridamole prevents lipid peroxidation and exhibits antioxidant properties in an *ex vivo* model [366]. Garćia-Fuentes et al. demonstrated in White Leghorn chicks that coconut oil-induced hypercholesterolemia was blunted with dipyridamole therapy [367]. Recently our group examined for the first time the role of dipyridamole in a mouse type 2 diabetic model to reduce oxidative stress as a protective mechanism of ischemia-induced angiogenesis during diabetes [368]. Dipyridamole therapy selectively and rapidly restores ischemic hind-limb blood flow in the diabetic mouse suggesting that it not only augments nitrite/NO endocrine functions but also directly reduces oxidative stress. There are earlier studies that showed altered blood glucose levels and maintenance of NO bioavailability following dipyridamole therapy [369–371]. Previous study by our group has shown that dipyridamole increases NO bioavailability by PKA dependent-eNOS pathway [364]. Another possible mode of dipyridamole action is through its antiplatelet effect. Therefore, antioxidant effect increased NO bioavailability and antiplatelets effect of dipyridamole improves endothelial dysfunction in both type of diabetes. Currently, metabolic effects and mode of action of dipyridamole therapy in diabetic vascular dysfunctions, such as diabetic retinopathy, are not known. Extensive studies in this line are much needed to understand the mechanistic aspects of dipyridamole during diabetes.

## 5. Conclusions

From the above discussion, it is obvious that endothelial dysfunction leading to defective angiogenesis in diabetes is multifactorial. Some of these factors are increased ROS and AGEs, decreased growth factors and cytokines, and altered immune cell responses. Similarly, defective diabetic wound healing is due to downregulation of different growth factors and overproduction of ROS leading to decreased NO bioavailability. On the other hand, excessive angiogenesis in diabetic retinopathy is multifactorial, as it involves increased growth factor and cytokine expression and increased oxidative stress, AGEs, and so forth. Researchers are trying to identify different agents that could provide vascular benefits from diabetes. Recent studies on therapies aimed at multiple factors of disease progression may act as an adjunct to the available conventional therapies. Improving clinical methodologies and techniques can further help in identifying the extent of endothelial damage, which could prevent the risk of disease progression. Studies aimed at combination therapies could prove beneficial to enhance protection against vascular complications during diabetes.

## Figures and Tables

**Table 1:** Comparison of aberrant angiogenesis under diabetes.

Defective angiogenesis		Excessive angiogenesis	
Phenotype	Causes	Phenotype	Causes

Reduced angiogenesis and collateral formation	Reduced VEGF, FGF, EPC circulation, cytokines, ECM/BM degradation; increased AGEs and MMP	Retinal capillary occlusion	Elevated intraocular pressure

Vascular occlusion, inflammation	Increased free fatty acids, polyol pathway, cytokines, ICAM, VCAM	Increased vascular permeability	Increased VEGF

Reduced wound healing; transplant failure	Reduced VEGF and growth factors; sorbitol-inositol imbalance; increased ACE, Ang-II and tissue factor mRNA	Capillary sprouting	Increased VEGF, FGF, PDGF; cytokines (TGF-*β*); integrins

Embryonic vasculopathy (anomalous vasculogenesis and angiogenesis)	Reduced VEGF, IL-1, TGF-*β*	Vascular remodeling	Increased laminin, fibronectin, collagen IV, ECM components, lipidosis
